# Performance of the TIMI risk score in predicting mortality after primary percutaneous coronary intervention in elderly women: Results from a developing country

**DOI:** 10.1371/journal.pone.0220289

**Published:** 2019-07-25

**Authors:** Shumaila Furnaz, Musa Karim, Tariq Ashraf, Sajjad Ali, Izza Shahid, Sara Ali, Uzzam Ahmed Khawaja, Muhammad Tanzeel ul Haque, Muhammad Shariq Usman, Tariq Jamal Siddiqi

**Affiliations:** 1 Department of Research, National Institute of Cardiovascular Diseases, Karachi, Pakistan; 2 Department of Medicine, Ziauddin Medical University, Karachi, Pakistan; 3 Department of Medicine, Aga Khan University, Karachi, Pakistan; 4 Department of Medicine, Jinnah Medical and Dental College, Karachi, Pakistan; 5 Department of Medicine, Dow University of Health Sciences, Karachi, Pakistan; Azienda Ospedaliero Universitaria Careggi, ITALY

## Abstract

**Background:**

Despite women undergoing primary percutaneous coronary intervention (PPCI) having a higher rate of adverse outcomes than men, data evaluating prognostic risk scores, especially in elderly women, remains scarce. This study was conducted to validate the predictive value of Thrombolysis in Myocardial Infarction (TIMI) risk score in elderly female patients.

**Materials and methods:**

This was a retrospective analysis of elderly (>65 years) female patients who underwent PPCI for ST-elevated myocardial infarction (STEMI) from October 2016 to September 2018. Patients’ demographic details and elements of TIMI risk score including age, co-morbidities, Killip classification; weight, anterior MI and total ischemic time were extracted from hospital records. The primary outcome was in-hospital mortality and post-discharge mortality reported on telephonic follow-up.

**Results:**

A total of 404 elderly women with a median age of 70 years were included. The mean TIMI score was 5.25±1.45 with 40.3% (163) patients of TIMI score > 5. In-hospital mortality rate was 6.4% (26) and was found to be associated with TIMI score (p<0.001). The in-hospital mortality rate increased from 3.1% at TIMI score of 0–4 to 34.6% at the score of 8. On follow-up (16.43±7.40 months) of 211 (55.8%) patients, the overall mortality rate was 20.3%, and this was also associated with TIMI score (p<0.001). The mortality rate increased from 5.6% at the score of 0–4 to 54.5% at the score of 8. The predictive values (area under the curve) of TIMI risk score for in-hospital and post-discharge mortality were 0.709 (95% CI 0.591–0.827; p <0.001) and 0.689 (95% CI 0.608–0.770; p <0.001), respectively.

**Conclusion:**

Increased adverse outcomes were observed with higher TIMI risk score for in hospital and post-discharge follow-up. Therefore, the prognostic TIMI risk score is a robust tool in predicting both in-hospital as well as post-discharge mortality in elderly females.

## Introduction

Primary percutaneous coronary intervention (PPCI) has played a significant role in reducing mortality rates in ST-segment elevation myocardial infarction (STEMI) patients [1]. Despite being the best management strategy with a decrease in number of re-infarctions [2], almost 10% of patients undergoing percutaneous coronary intervention (PCI) die within three years, [3] with women having a higher rate of adverse outcomes than men [4]. A recent multicentre registry that included STEMI patients undergoing PPCI from 12 countries showed a remarkably higher 30-day mortality rate in women, as compared to men [5]. Accurate post-PCI risk prediction in women is thus a necessity, to allow the delivery of appropriate therapeutic interventions to those at increased risk, and thus curtail mortality rates.

The ‘Thrombolysis in myocardial Infarction (TIMI) risk score’ is a risk stratification model which has been shown to accurately predict post-PCI mortality in both genders [6], with the c-statistic value of 30-day mortality ranging from 0.72–0.84 [7–9]. However, data evaluating the usefulness of the TIMI risk score in the South Asian population remains scarce. The validation of this risk score in the South Asian population is essential given the differences in genetics, lifestyle, and healthcare delivery in comparison to developed nations. Moreover, elderly patients with Acute Coronary Syndrome (ACS) are poorly analysed and underrepresented in clinical trials [10].

Given that 1 in 4 middle aged adults present with evidence of coronary artery disease (CAD) in Pakistan [11], we aimed to determine the predictive value of TIMI risk score and its prognostic significance; to profile the risk of mortality of elderly females who underwent PPCI in the largest ‘cardiac care center’ of the country.

## Methods

### Data source

This retrospective analysis was conducted in the department of adult cardiology, National Institute of Cardiovascular Diseases (NICVD), Karachi, Pakistan. Records of patients diagnosed with STEMI undergoing PPCI from October 2016 to September 2018 were obtained from the institutional database. Study was conducted after the approval of ethical review committee of NICVD (approval number: ERC-13/2019). Data in the database was anonymised before accessing them as per the institutional protocol. Written informed consent regarding the use of the use of a patient’s hospital records for research purposes was taken from all patients before the procedure. Data of patients who refused consent was excluded. Verbal consent was obtained at the time of telephonic follow-up.

### Study population

Female patients of > 65 years of age who were diagnosed with STEMI and subsequently underwent PPCI between October 2016 and September 2018 were included in the study. Only the first PPCI was included. Patients with missing data on patient sex, age, contact information, immediate outcome, in-hospital death or loss to follow-up were excluded from the analysis. Sample size for the study was calculated with an expected area under the curve (AUC) of 0.84 for TIMI risk score among women [7]. At 95% confidence level, and 5% margin of error, the minimum required sample size for the study was calculated to be 158 patients.

### Data collection/demographic details

Patients’ demographic details including age and sex, baseline characteristics, history of smoking, co-morbidities including history of documented hypertension or diabetes mellitus, positive family history of coronary artery disease, contact information and angiographic characteristics were recorded. Status of diabetes mellitus and hypertension was determined based on self-report or use of anti-diabetic or antihypertensive medication since past 6 months. A patient was classified as a ‘smoker’ if she acknowledged the habit at the time of enrolment, or had a history of smoking 10 or more cigarettes per day for at least 1 year. Follow up was obtained by contacting the patients or their relatives by telephone in order to record the study outcome variables.

### TIMI score

The following variables of the TIMI risk score were of concern: age, systolic blood pressure, diabetes, hypertension or angina, heart rate, Killip classification, weight, anterior MI and time to treatment. Time to treatment was defined as time from symptom onset to first balloon inflation. The calculation of the modified TIMI score is shown in Table 1 [6].

**Table 1:** Thrombolysis in myocardial infarction (TIMI) risk score calculation [6].

Serial	Variable	Level and score
1	Age	<65 years [0] 65 to 74 years [+2] ≥ 75 years [+3]
2	Diabetes, hypertension, or angina	No [0] Yes [+1]
3	Systolic blood pressure < 100 mmHg	No [0] Yes [+3]
4	Heart rate > 100 bpm	No [0] Yes [+2]
5	Killip Class II-IV	No [0] Yes [+2]
6	Weight < 67 kg	No [0] Yes [+1]
7	Anterior ST elevation or LBBB	No [0] Yes [+1]
8	Time to treatment > 4 hours	No [0] Yes [+1]

### Outcomes

The primary outcome of interest was the in-hospital mortality, defined as death of any cause within hospital stay of index procedure. A telephonic follow-up was made and self-reported (or attendant reported) incidence of post discharge mortality, re occurrence of MI, re-intervention, Coronary Artery Bypass Graft (CABG) surgery, and Transient Ischemic Attack (TIA) were recorded.

### Data analysis

IBM SPSS Statistics for Windows, Version 21.0. (IBM Corp., Armonk, NY, US) was used for the analysis of data. The Kolmogorov-Smirnov test was applied to the examine the normality of the continuous variables such as age (years), symptom onset to ER arrival time (min), ER to Lab (min), total Ischemic time (min), and TIMI risk score and appropriate mean ± SD or median [interquartile range (IQR)] were calculated. The categorical baseline and clinical characteristics were compared with study outcome by applying chi-square test, and the Mann-Whitney U test was performed to assess the TIMI risk score by study outcome. Univariate and multivariate logistic regression analysis were performed for the assessment of the association of TIMI risk score as well as other baseline characteristics of the patients, with post-procedure mortality. The results of the logistic regression analysis were expressed as an odds ratio (OR) along with 95% confidence interval (CI). The predictive value was determined using area under the curve (AUC) of receiver operating characteristic curve (ROC) of TIMI risk score with in-hospital mortality as state variable. The optimal cut-off value of TIMI risk score was determined by computing the Youden Index and patients were stratified based on optimal cut-off and in-hospital mortality and post discharge short term outcomes were compared between the patient groups. P-value less than or equal to 0.05 was considered statistically significant.

## Results

A total of 404 elderly (≥65 years) women undergoing PPCI for STEMI were included in this study. Median age of the patients was 70 [75–65] years with 29.2% (118) of the patients aged 75 years and above. Other than advanced age, other risk factors included hypertension (281 patients; 69.6%), diabetes (160 patients; 39.6%) and smoking (17 patients; 4.2%). The median time duration from symptoms onset to the arrival to emergency room was 240 [360–134.5] minutes and the median time from ER arrival to Cath lab activation was 70 [115–50] minutes. In 68.3% (276) patients, PPCI was performed after more than 4 hours of symptoms onset. More than two-thirds, 72.8% (294), of patients in our study were diagnosed with multi-vessel disease LAD occlusion in 49.8% (201) and RCA occlusion in 39.9% (161) patients. The mean TIMI score was 5 [6–4] with 40.3% (163) patients with TIMI score of more than 5 and post procedure in-hospital mortality rate of 6.4% (26). Risk profile and disease severity of the patient by post procedure in-hospital status are presented in Table 2.

**Table 2:** Risk profile and disease severity of elderly women underwent primary PCI for STEMI by in-hospital status.

Characteristics	Total	In-hospital Status		
		Survived	Expired	p-value
Total	404	378	26	-
**Risk Profile**				
Age (years)	70 [75–65]	70 [75–65]	70.5 [75–67]	0.18
Symptom onset to ER time (min)	240 [360–134.5]	240 [360–134]	246 [600–135]	0.329
ER to Lab (min)	70 [115–50]	70 [113–50]	73.5 [151–50]	0.617
Total Ischemic time (min)	320 [486–219.5]	314 [475–217]	370 [630–245]	0.316
65 to 74 years	286 (70.8%)	270 (71.4%)	16 (61.5%)	0.283
≥ 75 years	118 (29.2%)	108 (28.6%)	10 (38.5%)	0.283
Weight > 67 kg	227 (56.2%)	211 (55.8%)	16 (61.5%)	0.569
Killip class II-IV	67 (16.6%)	56 (14.8%)	11 (42.3%)	<0.001*
Diabetes	160 (39.6%)	147 (38.9%)	13 (50%)	0.262
Hypertension	281 (69.6%)	267 (70.6%)	14 (53.8%)	0.071
Smoking	17 (4.2%)	16 (4.2%)	1 (3.8%)	0.924
SBP< 100 mmHg	30 (7.4%)	21 (5.6%)	9 (34.6%)	<0.001*
Heart Rate (HR) > 100 bpm	50 (12.4%)	43 (11.4%)	7 (26.9%)	0.019*
Treatment time > 4 hours	276 (68.3%)	256 (67.7%)	20 (76.9%)	0.329
Anterior ST Elevation or LBBB	158 (39.1%)	148 (39.2%)	10 (38.5%)	0.944
TIMI Risk Score	5 [6–4]	5 [6–4]	6.5 [8–5]	<0.001*
**Disease Severity**				
**Number of vessels involved**				
Single vessel diseased (SVD)	110 (27.2%)	107 (28.3%)	3 (11.5%)	0.063
Two vessels diseased (2VD)	137 (33.9%)	128 (33.9%)	9 (34.6%)	0.937
Three vessels diseased (3VD)	157 (38.9%)	143 (37.8%)	14 (53.8%)	0.105
**Culprit Artery**				
LAD	201 (49.8%)	187 (49.5%)	14 (53.8%)	0.666
RCA	161 (39.9%)	153 (40.5%)	8 (30.8%)	0.328
CX	41 (10.1%)	37 (9.8%)	4 (15.4%)	0.36
LM	1 (0.2%)	1 (0.3%)	0 (0%)	0.792

p-values are computed based on chi-square test or Mann Whitney U test

*significant at 5%

Significantly higher proportion of patients who died during the index hospitalization, versus those who were discharged alive, had clinical presentation of Killip Class II-IV (42.3% (11) vs. 14.8%(56); p<0.001), systolic blood pressure (SBP) of less than 100 mmHg (34.6% (9) vs. 5.6%(21); p<0.001), and heart rate (HR) of more than 100 bpm (26.9% (7) vs. 11.4%(43); p<0.019). Moreover, the TIMI risk score was significantly higher among patients who did not survive as compared to those who survived (6.5 [8–5] vs. 5 [6–4]; p<0.001). The univariate and multivariate logistic regression analysis for in-hospital mortality are presented in Table 3.

**Table 3:** Predictors of in-hospital mortality.

Characteristics	Unadjusted		Adjusted	
	OR [95% CI]	p-value	OR [95% CI]	p-value
Age (years)	1.04 [0.98–1.1]	0.183	-	-
Total Ischemic time > 4 hours	1.59 [0.62–4.06]	0.333	-	-
Systolic blood pressure < 100 mmHg	9 [3.59–22.58]	<0.001*	5.06 [1.69–15.1]	0.004*
Heart Rate > 100 bpm	2.87 [1.14–7.22]	0.025*	1.46 [0.49–4.34]	0.497
Weight > 67 kg	1.27 [0.56–2.86]	0.57	-	-
KILLIP class II-IV	2.05 [1.36–3.11]	<0.001*	2.07 [0.62–6.93]	0.239
Diabetes	1.57 [0.71–3.48]	0.266	-	-
Hypertension	0.49 [0.22–1.08]	0.077	-	-
Smoking	0.91 [0.12–7.1]	0.924	-	-
Anterior ST Elevation or LBBB	0.97 [0.43–2.2]	0.944	-	-
Multivessel disease	3.03 [0.89–10.29]	0.076	-	-
TIMI Risk Score	1.71 [1.31–2.22]	<0.001*	1.17 [0.78–1.76]	0.452

Dependent variable: In-hospital mortality

*significant at 5%

**Fig 1:**
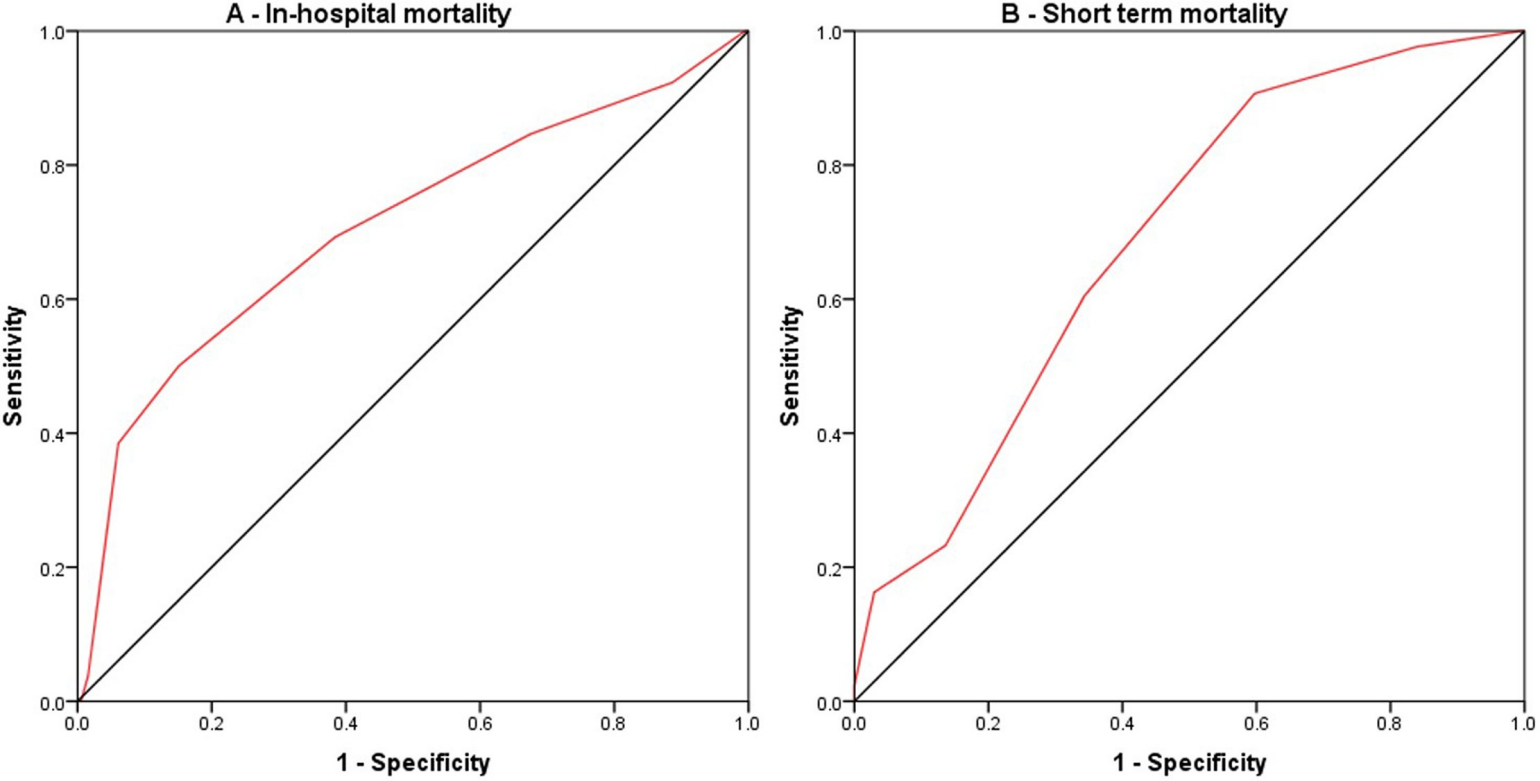
Receiver operating characteristic curve (ROC) for predictive value of TIMI Risk Score for A) In-hospital mortality and B) Short term mortality.

The predictive value, area under the curve (AUC) of receiver operating characteristic curve (ROC), of TIMI risk score for in-hospital mortality was found to be 0.709 (95% CI 0.591–0.827; p <0.001). Based on the Youden Index the optimal cut-off value of TIMI risk score was found to be 6 with sensitivity of 69.2%, specificity of 61.6%, and overall accuracy of 62.1%. The receiver operating characteristic curve (ROC) of TIMI risk score for in-hospital mortality and short-term mortality are presented in Fig 1.

**Fig 2:**
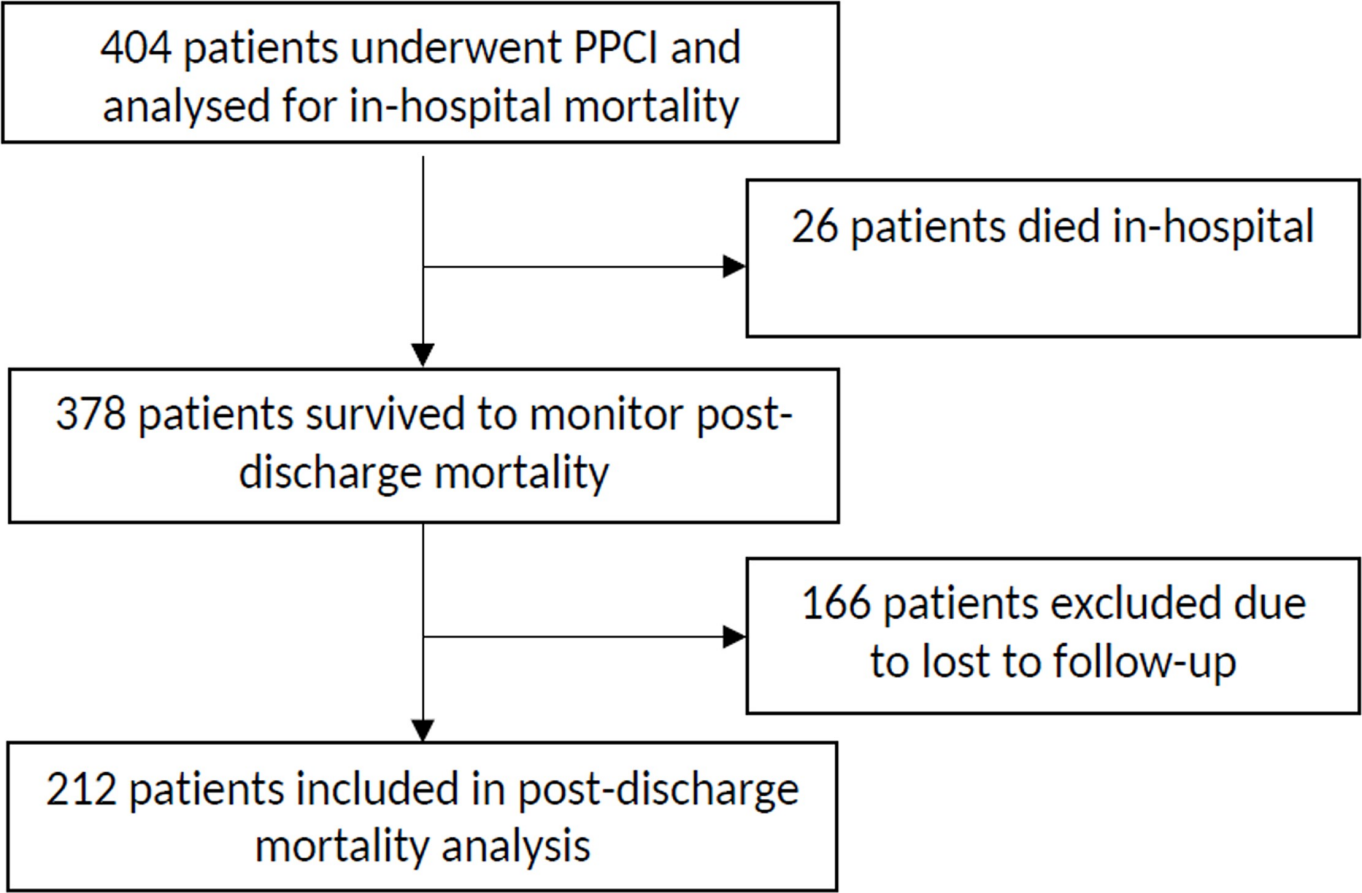
Flowchart of number of patients analyzed.

Telephonic follow-up was successfully conducted for 212 (56.1%) out of 378 survived patients (Fig 2) and the mean duration of follow-up was 16.43 ± 7.40 months ranging between 4 to 27 months. Outcomes included post discharge mortality rate of 20.3% (43/212), re occurrence of MI in 20.8% (44/212), re-intervention in 12.7% (27/212), CABG surgery in 5.7% (12/212), and transient ischemic attack (TIA) w 8.0% (17/212) of the patients.

Age and TIMI risk score were found to be independent predictors of post-discharge mortality with adjusted odds ratios of 1.1 [1.04–1.17] and 1.55 [1.18–2.05] respectively. The univariate and multivariate logistic regression analysis for post-discharge mortality are presented in Table 4.

**Table 4:** Predictors of post-discharge mortality.

Characteristics	Unadjusted		Adjusted	
	OR [95% CI]	p-value	OR [95% CI]	p-value
Age (years)	1.13 [1.06–1.19]	<0.001*	1.1 [1.04–1.17]	0.001*
Total Ischemic time > 4 hours	2.03 [0.91–4.51]	0.084	-	-
Systolic blood pressure < 100 mmHg	1.51 [0.38–5.95]	0.556	-	-
Heart Rate > 100 bpm	1.63 [0.63–4.2]	0.311	-	-
Weight > 67 kg	1.59 [0.8–3.16]	0.186	-	-
KILLIP class II-IV	1.83 [0.8–4.19]	0.153	-	-
Diabetes	1.1 [0.56–2.16]	0.791	-	-
Hypertension	0.99 [0.46–2.14]	0.985	-	-
Smoking	2.04 [0.49–8.5]	0.329	-	-
Anterior ST Elevation or LBBB	1.95 [0.99–3.84]	0.052	-	-
Multivessel disease	1.35 [0.62–2.94]	0.455	-	-
TIMI Risk Score	1.69 [1.3–2.19]	<0.001*	1.55 [1.18–2.05]	0.002*

Dependent variable: Post discharge mortality

*significant at 5%

Similarly, the predictive value, the area under the curve (AUC) of receiver operating characteristic curve (ROC), of TIMI risk score for post discharge mortality was found to be 0.689 (95% CI 0.608–0.770; p <0.001). The optimal cut-off value of 6 showed the sensitivity of 60.5%, specificity of 65.7%, and overall accuracy of 64.6%. The baseline TIMI risk score was found to be significantly associated with in-hospital as well as post discharge short term mortality (Chi-square test p-value <0.001). In-hospital mortality rate was 3.1% at TIMI score of 0–4 and rose up to 34.6% at the TIMI score of 8, however, due to a very small base size of seven patients in-hospital mortality rate at TIMI score of >8 was not aligned with the increment. Similarly, post discharge short term mortality rate was 5.6% at TIMI score of 0–4 and rose up to 54.5% at TIMI score of 8. In-hospital and post discharge mortality rate by baseline TIMI risk score are presented in Table 5.

**Table 5:** In-hospital and post discharge mortality rate by baseline TIMI risk score.

TIMI—Expected 30 Days Mortality		In-hospital Mortality		Post Discharge Mortality	
TIMI	Expected	Base (N)	Mortality (%)	Base (N)	Mortality (%)
0–4	0.8–7.3%	127	4 (3.1%)	72	4 (5.6%)
5	12%	114	4 (3.5%)	56	13 (23.2%)
6	16%	93	5 (5.4%)	51	16 (31.4%)
7	23%	37	3 (8.1%)	21	3 (14.3%)
8	27%	26	9 (34.6%)	11	6 (54.5%)
>8	36%	7	1 (14.3%)	1	1 (100%)
Total	13.5 ± 6.94%	404	26 (6.4%)	212	43 (20.3%)

Total expected 30 days mortality was calculated as average of expected 30 days mortality of individual patients

A similar association was again seen with a higher mortality rate among the patients with TIMI risk score ≥ 6 of 11.0% (18/163) vs. 3.3% (8/241), p = 0.001, in patients with TIMI risk score of less than 6. Similarly, post discharge mortality rate was 31% (26/84) in patients with score ≥6 vs. 13.3% (17/128), p = 0.001 in patients with TIMI score less than 6. Comparison of in-hospital mortality and post discharge short term outcomes by TIMI score-based grouping of patients was presented in Table 6.

**Table 6:** Comparison of in-hospital mortality and post discharge short term outcomes by TIMI score-based grouping of patients.

Outcomes	TIMI Risk Score		p-value
	TIMI 0–5	TIMI ≥ 6	
**Base**	**241**	**163**	-
In-hospital mortality	8 (3.3%)	18 (11.0%)	0.001*
**Post Discharge short term outcomes**			
**Base**	**128**	**84**	-
Mortality	17 (13.3%)	26 (31%)	0.001*
Re occurrence of MI	21 (16.4%)	23 (27.4%)	0.053
Re-intervention	15 (11.7%)	12 (14.3%)	0.583
CABG	9 (7%)	3 (3.6%)	0.286
Transient ischemic attack (TIA)	7 (5.5%)	10 (11.9%)	0.091

P-values are based on Chi-square Test

*significant at 5%

## Discussion

The burden of acute coronary syndrome is gaining increasing significance as the number of older population in Pakistan is gradually increasing. Mortality rate in elderly STEMI patients remains considerably higher as compared to younger patients [12], especially in women undergoing PPCI [7]. Evidence based therapies for elderly female STEMI patients are considerably underutilized, despite older women having worse prognosis than men [13,14].

Our current study evaluated in-hospital mortality as well as post discharge mortality rate. We analysed overall in-hospital mortality rate of 6.4% which is in contrast to the rate observed in previous studies (which ranges from 19% to 34%) [15–17]. Several variables were significantly related to in-hospital mortality such as Killip Class II-IV, Heart rate > 100bpm, systolic blood pressure <100 mmHg and TIMI risk score > 6 which was consistent with previous observations [7,18]. However, in contrast to previously published data [15], diabetes and hypertension were not significantly correlated to in-hospital mortality in the multivariate analysis.

Post discharge mortality rate at an average of 1 year was analysed to be 20.3% which is less than the rate reported from other similar studies (which range from 33 to 50.7% [19,20]). Almost one-fifth of those discharged suffered from another MI during the follow up period, suggesting that those with a baseline TIMI score of 6 or higher were likely to experience reoccurrence of MI and thus, have a greater likelihood of increased mortality.

We concluded that the TIMI risk score is highly predictive of in hospital and post-discharge mortality with a c-statistics of 0.709 and 0.689 respectively.

In our study, out of 404 patients who underwent PPCI, 40.3% (n = 163) were identified as high risk (TIMI Score ≥ 6) after the procedure. Stratification before the procedure, or while monitoring the follow up of high risk patients can also aid in deciding the appropriate intervention where an early triage may distinguish an invasive strategy which might significantly reduce mortality.

Thune et al. in the DANish trial in Acute Myocardial Infarction-2 (DANAMI-2) stratified patients according to TIMI score: 25% of patients were high risk (TIMI ≥5). After a 3 year follow-up, there was a remarkable reduction of mortality in patients who underwent primary PCI over thrombolytic therapy in high-risk group (25% mortality for PCI vs. 32.6% for fibrinolysis, p = 0.02) with no significant difference in low-risk patients (8% for PCI vs. 5.6% for fibrinolysis, p = 0.11) [22].

Moreover, the importance of stratification and TIMI score can also be judged from our analysis where post-discharge mortality was at least two times more (30.9%) in patients discerned as high risk when compared to those in the low risk strata (13.2%) further implying the need to have better targeted treatment and early management of those identified at an increased risk.

This confirms that TIMI risk score can be used as an early risk stratification tool to identify patients in which early therapeutic interventions could improve long term survival outcome, hence improving patient care. Since TIMI solely relies on objective assessment, it may particularly be important in female patients who often present with atypical symptoms, no chest pain and non‐diagnostic ECGs, making diagnosis difficult [21].

Furthermore, our findings can also be beneficial for clinicians where an existing validated risk tool provides an inexpensive method to evaluate patients. This is particularly useful for a developing country like Pakistan where majority of the population is catered by publicly funded health-care system which is already limited in scope [23].

### Limitations

Although bias was tried to be kept to a minimum, there are various limitations in this study that should be considered. Firstly, we did not analyse short term 30-day mortality. Comparing short term with long term post discharge mortality could have given a better prediction of the accuracy of TIMI risk score. Secondly, large cohorts from multiple settings are ideal to assess the validity of risk scores. Our analysis was only subjected to a small cohort from one cardiac care centre of the country therefore, our results may not be applicable to different hospital settings and patient population. Thirdly, loss-to-follow up data was large where post discharge mortality could only be compared in 212 patients as compared to the 404 patients that underwent PPCI. Fourthly, we only analysed all-cause mortality. Lastly, as no data regarding medication and adherence to the prescribed therapies were observed, therefore, potential confounding impact of these cannot be ruled out. Although TIMI score is validated for predicting all-cause mortality, further information about cause of death would have helped identify individual risk factors that may contribute to mortality in PPCI patients later in life.

## Conclusion

Our study validates the accuracy of TIMI risk score in elderly female patients by corroborating both in-hospital and long-term discriminative value, suggesting the use of this score in clinical setting to provide targeted treatment and monitor prognosis of a high-risk patient accordingly.

## Supporting information

S1 Dataset Dataset in SPSS format. (SAV)
